# Substrate stiffness modulates human gingival fibroblast paracrine signaling to promote osteogenic differentiation of human periodontal ligament cells 

**DOI:** 10.3389/fbioe.2026.1753774

**Published:** 2026-04-15

**Authors:** Watcharaphol Tiskratok, Masahiro Yamada, Maythwe Kyawsoewin, Hnin Yu Lwin, Hiroshi Egusa, Paiboon Jitprasertwong, Phoonsuk Limraksasin

**Affiliations:** 1 School of Geriatric Oral Health, Institute of Dentistry, Suranaree University of Technology, Nakhon Ratchasima, Thailand; 2 Center of Excellence for Dental Implantology, Oral Health Center, Suranaree University of Technology Hospital, Suranaree University of Technology, Nakhon Ratchasima, Thailand; 3 Division of Mechanobiology and Biomedical-Dental Engineering, Tohoku University Graduate School of Biomedical Engineering, Sendai, Miyagi, Japan; 4 Division of Molecular and Regenerative Prosthodontics, Tohoku University Graduate School of Dentistry, Sendai, Miyagi, Japan; 5 Center of Excellence for Dental Stem Cell Biology, Faculty of Dentistry, Chulalongkorn University, Bangkok, Thailand; 6 Department of Anatomy, Faculty of Dentistry, Chulalongkorn University, Bangkok, Thailand

**Keywords:** extracellular matrix stiffness, gingival fibroblasts, inflammatory responses, osteogenic differentiation, periodontal ligament cells

## Abstract

**Background:**

Human gingival fibroblasts (HGFs) are stromal cells that maintain periodontal tissue structure and extracellular matrix (ECM) dynamics. ECM stiffness serves as a physical cue that regulates HGF behavior and secretory profiles. This study investigated how substrate stiffness modulates the secretome of HGFs and observed the subsequent effects of this secretome on the osteogenic differentiation of human periodontal ligament cells (HPDLCs).

**Methods:**

HGFs isolated from healthy donors were cultured on polydimethylsiloxane substrates, representing soft or hard periodontal tissue under normal and lipopolysaccharide (LPS)-induced inflammatory conditions. The expression of cytokines and chemokines was analyzed using qRT-PCR and ELISA, with p38 MAPK inhibitors used to identify stiffness-associated signaling involvement. HPDLCs were treated with conditioned medium from HGFs (HGF-CM) under osteogenic induction, osteogenic marker expression was examined using qRT-PCR and immunofluorescence, with mineralization assessed by Alizarin red S staining. To establish mechanistic causality, functional blocking was conducted using a C-X-C motif chemokine receptor 4 (CXCR4) inhibitor.

**Results:**

Hard substrates significantly increased the expression of anti-inflammatory cytokines and the C-X-C motif chemokine ligand 12 (CXCL12) in HGFs, whereas inhibition of p38 mitogen-activated protein kinase (MAPK) activity attenuated stiffness-associated CXCL12 expression. Under LPS-induced inflammatory conditions, hard substrates-maintained matrix metalloproteinase-9 suppression and tissue inhibitor of metalloproteinases 1 upregulation, although CXCL12 protein levels were decreased. Furthermore, HPDLCs treated with HGF-CM derived from hard substrates in osteogenic induction media exhibited elevated *CXCR4* expression, increased osteogenic marker levels at days 14 and 21, and enhanced mineral deposition compared to those treated with HGF-CM from soft substrates. In addition, functional blocking with a CXCR4 inhibitor significantly reduced the expression of osteogenic markers (*ALP*, *RUNX2*, *COL1A1*, and *OSX*) and confirmed a subsequent decrease in matrix mineralization.

**Conclusion:**

Substrate stiffness modulated the paracrine behavior of HGFs, with CXCL12 serving as a representative example of a stiffness-responsive factor. These alterations in the HGF-derived secretome were associated with altered osteogenic and inflammatory responses in HPDLCs. These findings support the influence of the physical microenvironment on fibroblast–periodontal ligament cell interactions on anti-inflammatory response and periodontal tissue stabilization.

## 1 Introduction

Gingival fibroblasts (GFs) are the major stromal cell population in gingival connective tissues and are essential for maintaining tissue structure, extracellular matrix (ECM) turnover, and local immune responses. They synthesize and remodel collagen-rich matrices and respond to microbial and inflammatory cues by producing cytokines, chemokines, and matrix metalloproteinases (Naruishi, 2022; Wielento et al., 2023). The biomechanical properties of gingival tissues, largely determined by collagen organization and water content, are closely associated with periodontal health. Inflammation-driven alterations such as collagen degradation and tissue swelling markedly impact these mechanical properties (Moon et al., 2024), suggesting that gingival tissue elasticity may serve as a sensitive marker for potential indicator of inflammatory status of periodontal tissue.

Adjacent to the gingiva, periodontal ligament (PDL) fibroblasts represent a distinct stromal population critical for tooth support, tissue homeostasis and alveolar bone regeneration (Kim et al., 2013). Their dysregulation contributes to extracellular matrix breakdown, inflammatory signaling, and alveolar bone resorption in periodontitis. Although GFs and PDL fibroblasts occupy distinct anatomical niches in periodontium, their coordinated interactions through cytokine and chemokine secretion to support periodontal homeostasis and repair, highlighting the complexity of stromal biology within the periodontal microenvironment (Pi et al., 2007; Huang et al., 2024; Morandini et al., 2011).

Mechanical cues from the ECM are increasingly recognized as important regulators of fibroblast behavior (Gattazzo et al., 2014; Tiskratok et al., 2025). Matrix stiffness influences cell adhesion, proliferation, differentiation, and inflammatory mediator production through mechanotransduction pathways (Stanton et al., 2019). This feedback between fibroblasts and their mechanical microenvironment is well described in various biological processes, including wound healing and fibrosis. In oral tissues, ECM stiffness has been shown to modulate proinflammatory responses in gingival fibroblasts and to regulate the osteogenic differentiation of human periodontal ligament cells (HPDLCs) (Liu et al., 2018; Ding et al., 2025). Furthermore, gingival fibroblasts also secrete a diverse repertoire of paracrine factors that influence the behavior of neighboring cells. Interestingly, the chemokine CXCL12, also known as stromal-cell derived factor-1 (SDF-1), is constitutively expressed by fibroblasts, including gingival and periodontal ligament fibroblasts, and is known to regulate cell migration, survival, and osteogenic differentiation via CXCR4-mediated signaling (Hosokawa et al., 2005; Yashiro et al., 2014; Yellowley, 2013; Liu et al., 2013). However, how ECM stiffness regulates the secretory profile of human gingival fibroblasts, and how these stiffness-dependent paracrine signals influence HPDLC osteogenesis, remain unclear.

Therefore, this study aims to investigate the influence of ECM stiffness on paracrine behavior of human gingival fibroblasts and its subsequent effect on the osteogenic differentiation of human periodontal ligament cells, with a particular focus on stiffness-responsive chemokine secretion as a representative example of these paracrine interactions. By elucidating the relationship between ECM mechanics, fibroblast-derived chemokines, and osteogenic differentiation, this research seeks to provide the interplay between biomechanical microenvironment on fibroblast-mediated anti-inflammatory responses for periodontal tissue stabilization.

## 2 Materials and methods

### 2.1 Preparation of culture substrate stiffness

A vinyl-terminated base and methyl hydrogen siloxane curing agent of commercial PDMS (Sylgard 527; Dow Corning, NY, USA) were prepared according to a previously reported method (Razafiarison et al., 2018). To fabricate substrates with varying stiffness levels, the vinyl-terminated base was mixed with the methyl hydrogen siloxane curing agent in weight ratios of 5:4 and 4:5, resulting in soft (4.4 kPa) and hard (26.2 kPa) substrates, respectively. These mixtures were then polymerized at 65 °C for 4 h under controlled condition. To prepare the culture surfaces, either polystyrene culture plates or sterilized PDMS substrates were coated with oxygen plasma and 0.01 wt% bovine dermis-derived native type I collagen solution (IAC-30; Koken Co., Ltd., Tokyo, Japan). The plates were then incubated at room temperature for 90 min to facilitate proper collagen adsorption.

### 2.2 Human gingival fibroblasts (HGFs) cultures

HGFs were isolated from gingival tissue obtained from five periodontally healthy donors (18–25 years) who came to the Oral Health Centre, Suranaree University of Technology Hospital. Tooth extractions were performed either due to impaction or for orthodontic reasons. The isolation and cultivation of HGFs followed established protocols from prior research (Egusa et al., 2010). All participants provided written informed consent form prior to participation, and the study protocol was approved by the Ethics Committee of Suranaree University of Technology (approval no. EC-68-78). HGFs were cultured in a high-glucose Dulbecco Modified Eagle Medium (DMEM) (Gibco, Grand Island, NY, USA), supplemented with 10% fetal bovine serum (FBS) (Gibco), 2 mM L-glutamine (Gibco), 100U/mL penicillin (Gibco),100 mg/mL streptomycin (Gibco), and 5 mg/mL amphotericin B (Gibco) at 37 °C in a 5% CO_2_ atmosphere. After 80% confluence, the cells were detached using 0.25% trypsin/1 mM ethylenediaminetetraacetic acid (Gibco) and seeded in the HGF growth media on collagen-coated substrates of a 12- or 24-well culture-graded polystyrene plate or the soft, or hard PDMS. The medium was renewed every 48 h, and passages three to seven were utilized in all the experiments conducted in this study.

The culture supernatants of HGFs cultured for 24 h on each substrate were collected and stored at −80 °C prior to use in the subsequent co-culture experiment.

### 2.3 Human periodontal ligament cells (HPDLCs) culture

Human periodontal ligament tissues were collected from periodontally healthy molars of five healthy donors. HPDLCs were isolated and cultured according to methods described in a previous report (Kanjanamekanant et al., 2014). All participants provided written informed consent form prior to participation, and the study protocol was approved by the Ethics Committee of Suranaree University of Technology (approval no. EC-66-59). In summary, the periodontal ligament was scraped from the middle third of the roots of molars and placed in a culture plate for 3–4 weeks in a high-glucose DMEM (Gibco), supplemented with 10% FBS (Gibco), 2 mML-glutamine (Gibco), 100U/mL penicillin (Gibco),100 mg/mL streptomycin (Gibco), and 5 mg/mL amphotericin B (Gibco). The cells were incubated at 37 °C in a humidified atmosphere with 5% CO_2_, and the culture medium was changed every 48 h. Cells were subcultured at a 1:3 ratio using 0.25% trypsin EDTA (Gibco) once they reached 80% confluency, and Passages three to seven were utilized in all the experiments conducted in this study.

For osteogenic differentiation, the cells were cultured in an osteogenic induction medium containing a growth medium supplemented with 250 nM dexamethasone (Sigma-Aldrich, USA), 50 μg/mL ascorbic acid (Sigma-Aldrich, USA), and 5 mM β-glycerophosphate (Sigma-Aldrich, USA).

### 2.4 HPDLCs coculture with conditioned medium of HGFs

HPDLCs were initially cultured in an osteogenic induction media on 12- or 24-well polystyrene culture plates for 24 h. Subsequently, the medium was replaced with osteogenic induction medium supplemented with 10% (v/v) HGF-conditioned medium (HGF-CM) derived from HGFs cultured on polystyrene, soft, or hard substrates. To prevent dilution effects, the final concentrations of FBS and osteogenic supplements were adjusted to remain constant across all experimental and control groups. The HPDLCs were maintained in these conditions for 24 h, 14 days, or 21 days, respectively.

### 2.5 Inhibition of CXCR4

HPDLCs were initially cultured in an osteogenic induction media on 24-well polystyrene culture plates for 24 h. Following this, the HPDLCs were pretreated with 100 ng/mL of CXCR4 antagonist (AMD-3100) (Sigma-Aldrich, St. Louis, MO, USA) (L et al., 2017) for 1 h. After pre-treatment, the HPDLCs were cultured with conditioned medium from HGFs on hard stiffness for 21 days.

### 2.6 Induction of inflammatory responses

HGFs were cultured on collagen-coated PDMS substrates placed in 24-well polystyrene plates and incubated in growth medium containing 1 μg/ml of *P. gingivalis (Porphyromonas gingivalis)* LPS (Sigma-Aldrich, St. Louis, MO, USA) (Park and Yoon, 2022). The cells were incubated for 24 h.

### 2.7 Inhibition of cellular mechanotransduction

HGFs were cultured on collagen-coated PDMS with hard stiffness at cell density of 6 × 10^4^ cells per well in a 24-well culture plate, with polystyrene used as a control. The cells were then incubated at 37 °C with 5% CO_2_. Following incubation for 24 h, the groups subjected to hard stiffness underwent treatment with 1.5 nM ERK inhibitor (Calbiochem®, Merck KGaA, Darmstadt, Germany) (Suwittayarak et al., 2022) or 35 nM inhibitor of p38 MAPK (SB203580, 35nM, cat. No. 506126, Calbiochem®) (Manokawinchoke et al., 2019) for 3 h prior to sample collection. CXCL12 gene and protein expression were then analyzed.

### 2.8 Reverse transcription–polymerase chain reaction (RT–PCR)

RNA isolation was done using RiboEx total RNA isolation solution (GeneAll, Seoul, Korea). 1 μg of total RNA was converted to into complementary DNA utilizing the ImProm-II Reverse Transcription System from Promega (Madison, WI, USA), after which it underwent real-time PCR analysis. The cycling protocol consisted of an initial step at 95 °C for 30 s, followed by 40 cycles of 95 °C for 3 s and 60 °C for 30 s. Real-time RT-PCR was conducted using the Green Master Mix (Promega Corporation, Madison, WI, USA) on a Roche real-time PCR system, adhering to the manufacturer’s guidelines. The expression levels of target genes were quantitatively assessed through the comparative cycle time (ΔΔCT) method, with Glyceraldehyde 3-phosphate dehydrogenase (GAPDH) serving as the housekeeping gene. The primer sequences utilized for the real-time RT-PCR are documented in (Table 1).

**TABLE 1:** Sequences of primers used for real-time reverse transcription PCR (RT-PCR).

Gene name	Primer sequence 5′-3′	Product size (bp)	Accession number
*GAPDH*	(F) CAC TGC CAA CGT GTC AGT GGT G	121	NM_001289746.2
	(R) GTA GCC CAG GAT GCC CTT GAG		
*IL4*	(F) TCT GTG CAC CGA GTT GAC C	127	NM_000589.4
	(R) GCG AGT GTC CTT CTC ATG GT		
*IL10*	(F) TGC TCT TGC AAA ACC AAA CCA	171	NM_000572.3
	(R) TCG AAG CAT GTT AGG CAG GTT		
*IL1B*	(F) GCA GAA GTA CCT GAG CTC GC	174	NM_000576.3
	(R) CTT GCT GTA GTG GTG GTC GG		
*IL6*	(F) AGA CAG CCA CTC ACC TCT TCA G	1127	NM_000600.1
	(R) TTC TGC CAG TGC CTC TTT GCT G		
*TNFA*	(F) CTC TTC TGC CTG CAC TTT G	1678	NM_000594.4
	(R) ATG GGC TAC AGG CTT GTC ACT C		
*MMP9*	(F) CAG TCC ACC CTT GTG CTC TT	120	NM_004994.3
	(R) CGA CTC TCC ACG CAT CTC TG		
*MMP8*	(F) AAC CAC CAC TTC CTC TCC TG	129	NM_001304442.2
	(R) ATA GTG TGT GCC CTC CTG AG		
*TIMP1*	(F) GGA GAG TGT CTG CGG ATA CTT C	100	NM_003254.3
	(R) GCA GGT AGT GAT GTG CAA GAG TC		
*CXCL12*	(F) CAC AGA AGG TCC TGG TGG TA	249	NM_001178134.2
	(R) CAT TGA AAA GCT GCA ATC ACA		
*CXCR4*	(F) GCA GGT AGC AAA GTG ACG C	83	NM_003467.3
	(R) GAT CCC CTC CAT GGT AAC CG		
*ALP Alkaline phosphatase, biomineralization associated (ALPL)*	(F) GCT​GTA​AGG​ACA​TCG​CCT​ACC​A	131	NM_000478.6
	(R) CCT​GGC​TTT​CTC​GTC​ACT​CTC​A		
*RUNX2*	(F) ATG ATG ACA CTG CCA CCT CTG A	167	NM_001369405.1
	(R) GGC TGG ATA GTG CAT TCG TG		
*COL1A1*	(F)GTG CTA AAG GTG CCA ATG GT	128	NM_000088.4
	(R) ACC AGG TTC ACC GCT GTT AC		
*OPN* *Secreted Phosphoprotein 1 (SPP1)*	(F) AGG AGG AGG CAG AGC ACA	150	NM_001251830.2
	(R) CTG GTA TGG CAC AGG TGA TG		
*OCN (PMF1-BGLAP)*	(F) CTT TGT GTC CAA GCA GGA GG	166	NM_001199663.1
	(R) CTG AAA GCC GAT GTG GTC AG		
*OSX* *Sp7 transcription factor (SP7)*	(F) GCC AGA AGC TGT GAA ACC TC	161	NM_001173467.3
	(R) GCT GCA AGC TCT CCA TAA CC		

*GAPDH*, Glyceraldehyde-3-phosphate dehydrogenase; *IL4*, Interleukin-4; *IL10*, Interleukin-10; IL1B, Interleukin-1, beta; *MMP9*, Matrix metallopeptidase 9; *MMP8,* Matrix metallopeptidase 8; *TIMP1*, Tissue inhibitor of metalloproteinases 1; *CXCL12*, C-X-C motif chemokine ligand 12; *CXCR4*, C-X-C motif chemokine receptor 4; *ALP*, alkaline phosphatase; *RUNX2*, Runt-related transcription factor 2; *COL1A1*, Collagen type I alpha 1 chain; *OPN*, osteopontin; *OCN*, osteocalcin; *OSX*, osterix.

### 2.9 Immunofluorescence staining

The cells on polystyrene culture plates and PDMS were fixed with a 4% paraformaldehyde phosphate buffer solution (FUJIFILM Wako Pure Chemical Corporation) for 15 min. Following fixation, the cells were washed with PBS and subsequently blocked to prevent non-specific protein binding using a blocking buffer comprising of 2.0% bovine serum albumin (BSA) (Sigma-Aldrich), 0.1% Triton-X (USBiological Life Sciences), and 0.01% Tween 20 (Sigma-Aldrich) for 1 hour. For the detection of specific cellular markers, the cells underwent fixation, permeabilization, and blocking for non-specific proteins, followed by overnight incubation at 4 °C with a primary antibody with CXCR4 monoclonal antibody (ab 124,824, 1:50 dilution in 1% BSA, abcam, Cambridge, UK) and anti-osterix monoclonal antibody (F-3, sc-393325: 1:50 dilution in 1% BSA, Santa Cruz Biotechnology, Dallas, TX, USA). Following this incubation, the samples were exposed to an Alexa Fluor 488-conjugated goat anti-mouse IgG (diluted 1:1000 in 1% BSA, Molecular Probes, Thermo Fisher Scientific) for 1 hour at room temperature. The samples were also treated with Rhodamine-phalloidin (diluted 1:1000 in 1% BSA, Cytoskeleton), using PBS as the diluent. After washing the samples with PBS, they were mounted onto slides using an anti-fade mounting medium containing DAPI (Vectashield, Vecta Laboratory, USA). Immunofluorescent microscopy and analyses were conducted using an apotome fluorescent microscope (Axio Observer Z1 and ZEN pro, ZEISS International, Oberkochen, Germany).

### 2.10 Alizarin red S (ARS) staining

ARS staining was evaluated after 14 days and 21 days of incubation in osteogenic induction medium. Prior to fixation with 4% paraformaldehyde in phosphate buffer, the cells were rinsed with phosphate-buffered saline (PBS). Subsequently, the cells were incubated in a 2% Alizarin red S solution (Sigma) at a pH range of 4.1–4.3 for 20 min, then washed with distilled water, and allowed to dry before capturing images under a light microscope.

The mineral deposits were dissolved with 10% cetylpyridinium chloride monohydrate (Sigma-Aldrich, St. Lousi, MO, USA) in phosphate buffer, for quantitative analysis. The solution was transferred to a 96-well plate and the optical density was measured at 570 nm with a microplate reader.

### 2.11 Quantification of CXCL12 using enzyme-linked immunosorbent assay (ELISA)

CXCL12 protein expression levels were determined by ELISA. CXCL12 protein in conditioned medium was measured by Human CXCL12/SDF-1α ELISA Kit (Quantikine, R&D systems. Inc., Minneapolis, USA). The procedure was performed according to the manufacturer’s instructions.

### 2.12 Statistical analysis

Statistical analyses were conducted using IBM SPSS Statistics for Windows, version 29 (IBM Corp., Armonk, NY, USA). One-way or two-way ANOVA was performed with subsequent Tukey’s multiple comparison tests. A *P* value of less than 0.05 was considered statistically significant. Sample sizes are described in the figure legends. As specified in the figure legends, *n* represents the number of independent biological replicates derived from separate donors.

## 3 Results

### 3.1 Substrate stiffness induced the expression of anti-inflammatory cytokines, matrix metalloproteinases, and CXCL12 in HGFs

**FIGURE 1:**
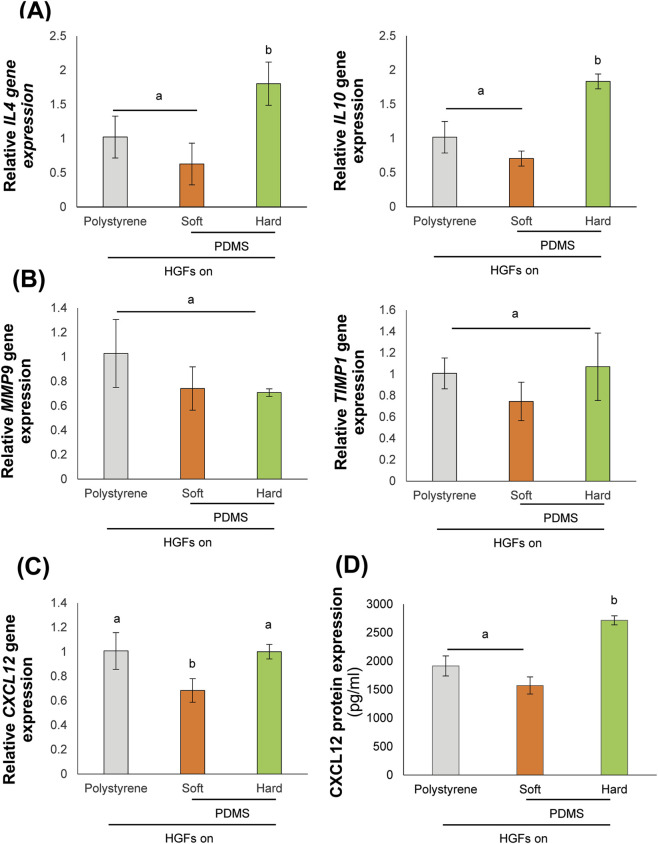
Substrate stiffness regulates the behaviors of human gingival fibroblasts (HGFs). Real-time reverse transcription-polymerase chain reaction (RT-PCR) was performed to detect gene expression levels of **(A)** anti-inflammatory markers, *IL4*, and *IL10*, **(B)** matrix metalloproteinase markers, including *MMP9*, and *TIMP1*, **(C)** chemokine, *CXCL12*. The expression of *GAPDH* was used as an internal control. Data were statistically analyzed by one-way ANOVA followed by Tukey’s multiple comparison tests (*n* = 3: *P* < 0.05). **(D)** The expression of GAPDH was used as an internal control. Data were statistically analyzed by one-way ANOVA followed by Tukey’s multiple comparison tests (*n* = 3: P < 0.05). **(D)** The protein expression of CXCL12 was detected by ELISA analysis. Data were statistically analyzed by one-way ANOVA followed by Tukey’s multiple comparison tests (*n* = 4: P < 0.05). Data are presented as the mean ± standard deviation (SD), with different letters indicating statistically significant differences between multiple groups. IL4, interleukin 4; IL10, interleukin 10; MMP9, matrix metalloproteinase 9; TIMP1, tissue inhibitor of matrix metalloproteinases 1; CXCL12, CXC motif chemokine 12; GAPDH, glyceraldehyde-3-phosphate dehydrogenase; PDMS, polydimethylsiloxane.

Substrate stiffness regulated the gene expression of HGFs after 24 h. The expression of anti-inflammatory cytokine markers, *IL4* and *IL10*, was significantly increased on the hard substrate compared to both polystyrene and the soft substrates (Figure 1A). There was no significant difference in *MMP9* gene expression between HGFs cultured on soft and hard PDMS substrates. In addition, *TIMP1* expression did not show significant variation across the different substrates (Figure 1B). *CXCL12* gene expression was significantly reduced on soft substrates but remained at higher levels on polystyrene and hard substrates (Figure 1C). Protein analysis confirmed a higher concentration of CXCL12 in the conditioned medium of HGFs cultured on the hard substrate (Figure 1D), indicating that substrate stiffness influences the expression of anti-inflammatory cytokines, matrix metalloproteinases, and CXCL12.

### 3.2 Substrate stiffness regulated cytokine and chemokine expression of HGFs under an inflammatory condition

**FIGURE 2:**
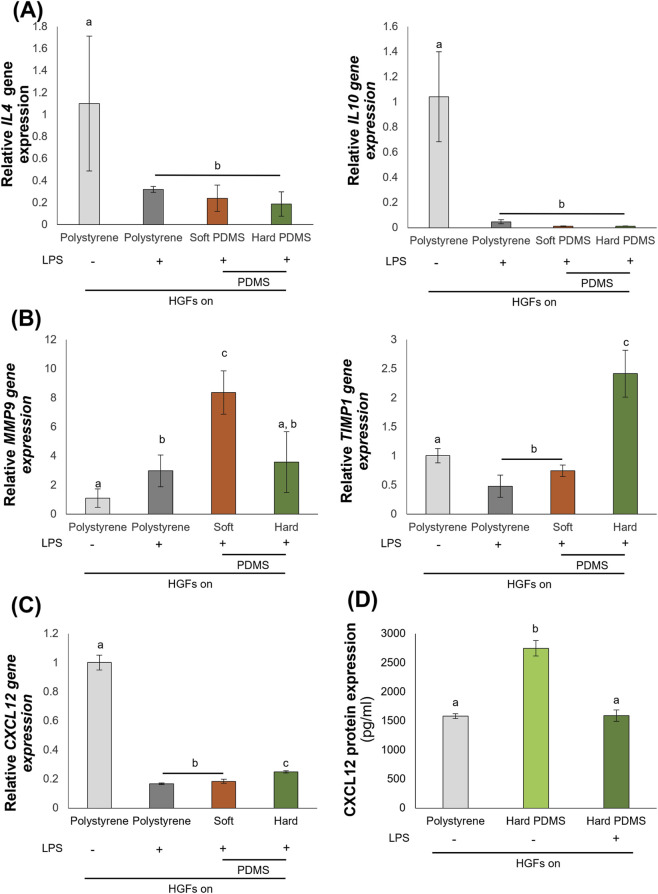
Substrate stiffness regulates human gingival fibroblasts (HGFs) behaviors under an inflammatory condition. Real-time RT-PCR was performed to detect gene expression levels of **(A)** *IL4* and *IL10*, **(B)** *MMP9* and *TIMP1*, and **(C)** *CXCL12*. The expression of *GAPDH* was used as an internal control. Data were statistically analyzed by one-way ANOVA followed by Tukey’s multiple comparison tests (*n* = 3: P < 0.05). **(D)** The expression protein of CXCL12 was detected by ELISA analysis. Data were statistically analyzed by one-way ANOVA followed by Tukey’s multiple comparison tests (*n* = 4: P < 0.05). Data are presented as the mean ± standard deviation (SD), with different letters indicating statistically significant differences between multiple groups. IL4, interleukin 4; IL10, interleukin 10; MMP9, matrix metalloproteinase 9; TIMP1, tissue inhibitor of matrix metalloproteinases 1; CXCL12, CXC motif chemokine 12; GAPDH, glyceraldehyde-3-phosphate dehydrogenase; PDMS, polydimethylsiloxane; LPS, lipopolysaccharide.

HGFs cultured on substrates with LPS treatment for 24 h showed no significant differences in *IL4* and *IL10* expression between soft and hard PDMS (Figure 2A). However, *MMP9* gene expression was significantly decreased, while *TIMP1* expression was significantly increased on the hard substrate compared to the soft substrate (Figure 2B). Interestingly, *CXCL12* gene expression was upregulated in HGFs on the hard substrate compared with those on the soft substrate (Figure 2C). In addition, ELISA results confirmed that CXCL12 protein levels were significantly reduced following LPS treatment (Figure 2D), indicating an inhibitory effect of inflammation on CXCL12 protein expression.

### 3.3 Involvement of p38 mitogen-activated protein kinase (MAPK) activity in stiffness-associated CXCL12 expression in HGFs

**FIGURE 3:**
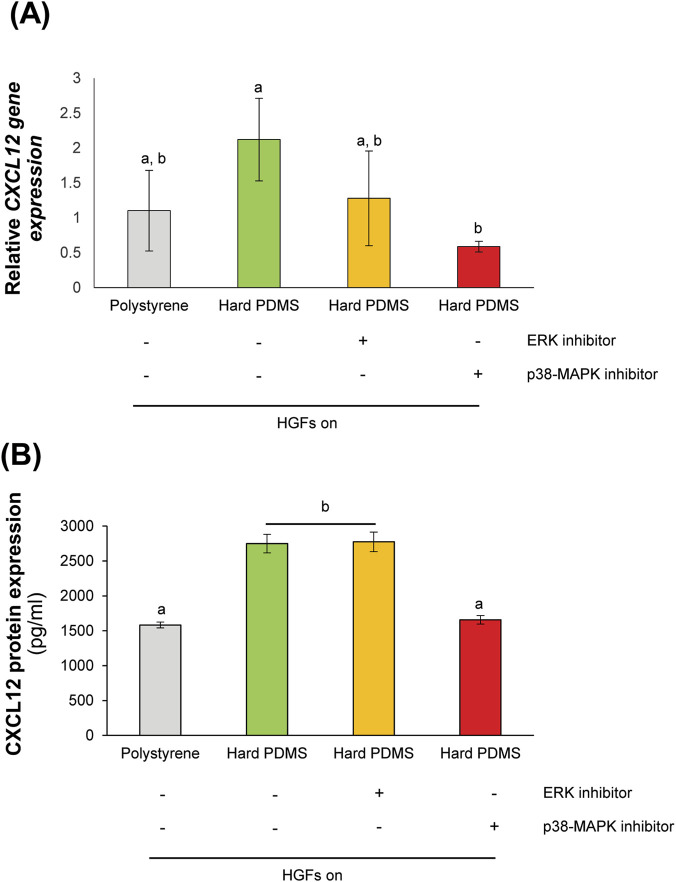
Mitogen-activated protein kinase (MAPK) pathway regulated substrate stiffness-induced CXCL12 expression in human gingival fibroblasts (HGFs). **(A)** Real-time RT-PCR was performed to detect gene expression levels of *CXCL12*. The expression of *GAPDH* was used as an internal control. Data were statistically analyzed by one-way ANOVA followed by Tukey’s multiple comparison tests (*n* = 3: *P* < 0.05). **(B)** The protein expression of CXCL12 was detected by ELISA analysis. Data were statistically analyzed by one-way ANOVA followed by Tukey’s multiple comparison tests. (*n* = 4: *P* < 0.05). Data are presented as the mean ± standard deviation (SD), with different letters indicating statistically significant differences between multiple groups. CXCL12, CXC motif chemokine 12; GAPDH, glyceraldehyde-3-phosphate dehydrogenase; PDMS, polydimethylsiloxane.

The signaling mechanisms associated with substrate stiffness–induced CXCL12 expression were investigated. HGFs were treated with p-38 or ERK inhibitor for 3 h. The results demonstrated that CXCL12 gene and protein expression were significantly decreased in HGFs on the hard substrate following p38 MAPK inhibitor treatment (Figures 3A,B). In contrast, ERK inhibition did not significantly affect CXCL12 expression compared to untreated controls on the hard substrate, suggesting that p38 MAPK activity might be associated with stiffness-responsive regulation of CXCL12 expression in HGFs.

Furthermore, p38 MAPK inhibition significantly suppressed the expression of the anti-inflammatory cytokine IL4 in HGFs on hard substrates (Supplementary Figure S1A). Conversely, the gene expression of pro-inflammatory cytokines, specifically *IL1B*, *IL6*, and *TNF*, was markedly upregulated following the blockade of p38 activity (Supplementary Figure S1B). These results suggest that p38 MAPK signaling not only drives CXCL12 production but also associated to regulate inflammatory responses in HGFs.

### 3.4 Effects of conditioned media of HGFs (HGF-CM) under different substrate stiffness on HPDLCs’ behaviors

**FIGURE 4:**
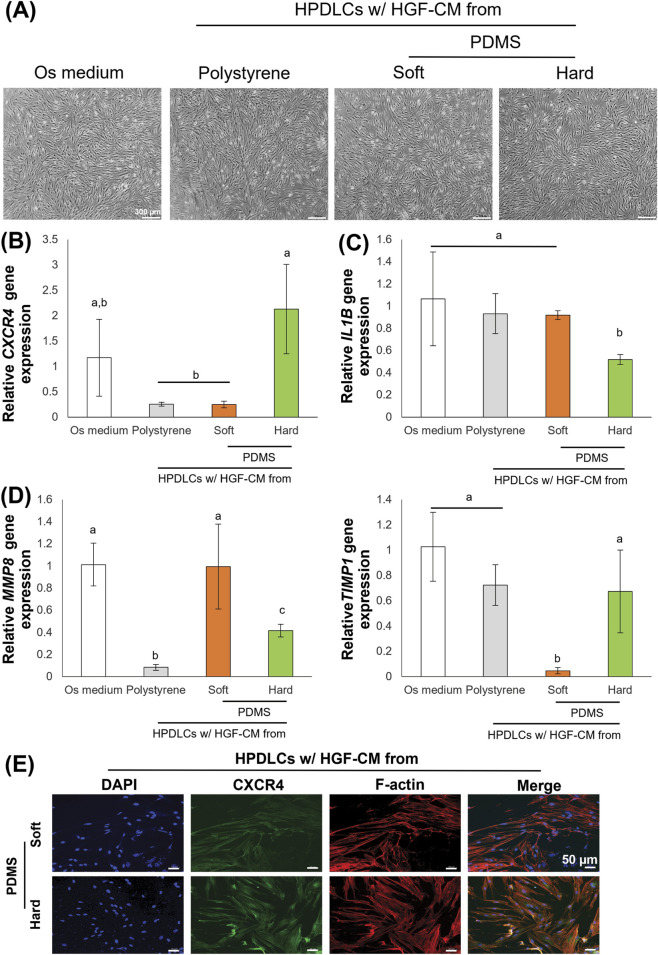
Effects of conditioned media of human gingival fibroblasts (HGF-CM) under different substrate stiffness on human periodontal ligament cells’ behaviors. **(A)** Human periodontal ligament cells (HPDLCs) cultured with HGF-CM in osteogenic medium for 24 h. HPDLCs morphology was demonstrated using a phase-contrast microscope. Scale bars: 300 μm. **(B)** Real-time RT-PCR was performed to detect gene expression levels of *CXCR4*, which is receptor of CXCL12. **(C)** Real-time RT-PCR was performed to detect gene expression levels of pro-inflammatory cytokine, *IL1b*. **(D)** Real-time RT-PCR was performed to detect gene expression levels of *MMP8* and *TIMP1*. The expression of GAPDH was used as an internal control. **(E)** Immunofluorescence analysis was performed to detect the protein expression of CXCR4 (green). The cytoskeleton (F-actin; red) and nuclei (blue) were stained using rhodamine-phalloidin and DAPI, respectively. Scale bars: 50 μm. Data were statistically analyzed by one-way ANOVA followed by Tukey’s multiple comparison tests (*n* = 3: P < 0.05). Data are presented as the mean ± standard deviation (SD), with different letters indicating statistically significant differences between multiple groups. IL1B, interleukin-1β; MMP8, matrix metalloproteinase 8; TIMP1, tissue inhibitor of matrix metalloproteinases 1; CXCR4, CXC motif receptor type 4; GAPDH, glyceraldehyde-3-phosphate dehydrogenase; PDMS, polydimethylsiloxane.

HPDLCs cultured in osteogenic medium treated with HGF-CM for 24 h did not show altered cell morphology compared to those cultured in osteogenic medium (Figure 4A). HGF-CM from the hard substrate group showed significantly increased *CXCR4* expression compared to polystyrene and soft substrate groups (Figure 4B). This finding was further evaluated by immunofluorescence staining, which revealed markedly higher CXCR4 protein intensity in HPDLCs treated with hard-substrate HGF-CM compared to those treated with soft-substrate HGF-CM (Figure 4E). The expression of the proinflammatory cytokine *IL1B* was reduced in HPDLCs with HGF-CM from the hard substrate (Figure 4C). Regarding matrix remodeling, HPDLCs exposed to HGF-CM from the hard substrate showed decreased *MMP8* expression, while *TIMP1* expression was upregulated (Figure 4D).

### 3.5 Effects of HGF-CM on substrate stiffness regulated osteogenic differentiation of HPDLCs

**FIGURE 5:**
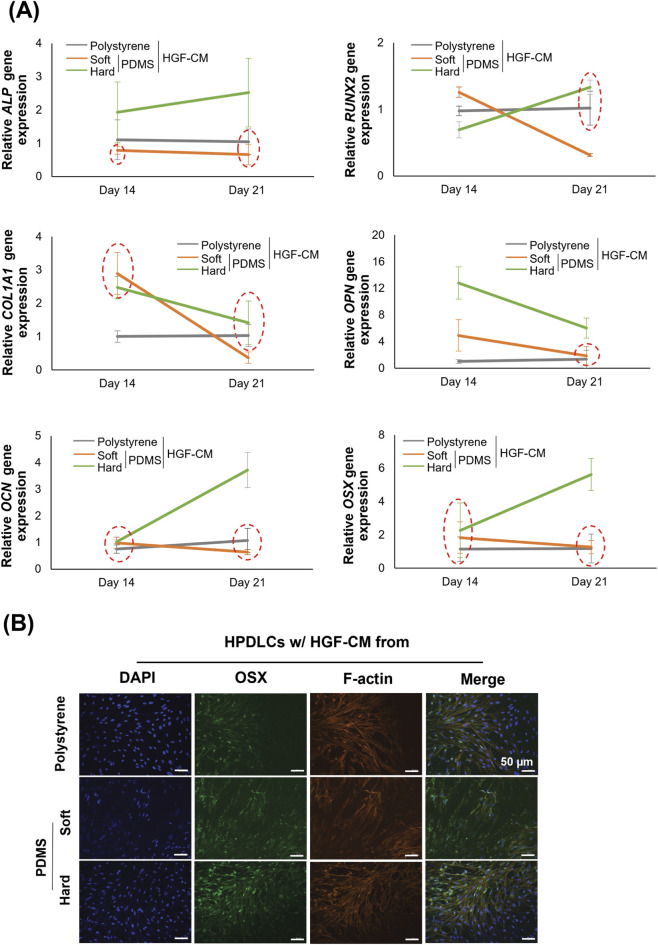
Effects of conditioned media of human gingival fibroblasts (HGF-CM) on substrate stiffness regulated osteogenic differentiation of human periodontal ligament cells (HPDLCs) on day 14 and day 21. **(A)** Real-time RT-PCR was performed to detect gene expression levels of osteogenic markers, including *ALP, RUNX2, COL1A1, OPN, OCN*, and *OSX*. The expression of GAPDH was used as an internal control. Data were statistically analyzed by two-way ANOVA followed by Tukey’s multiple comparison tests. (*n* = 3: P < 0.05). **(B)** Immunofluorescence analysis was performed to detect the protein expression and localization of OSX (green). The cytoskeleton (F-actin; red) and nuclei (blue) were stained using rhodamine-phalloidin and DAPI, respectively. Scale bars: 50 μm. Data are presented as the mean ± standard deviation (SD), with red dotted circle indicating no statistically significant differences between multiple groups. ALP, alkaline phosphatase; RUNX2, runt-related transcription factor 2; COL1A1, collagen type I alpha 1; OPN, osteopontin; OCN, osteocalcin; OSX, osterix; GAPDH, glyceraldehyde-3-phosphate dehydrogenase; PDMS, polydimethylsiloxane.

**FIGURE 6:**
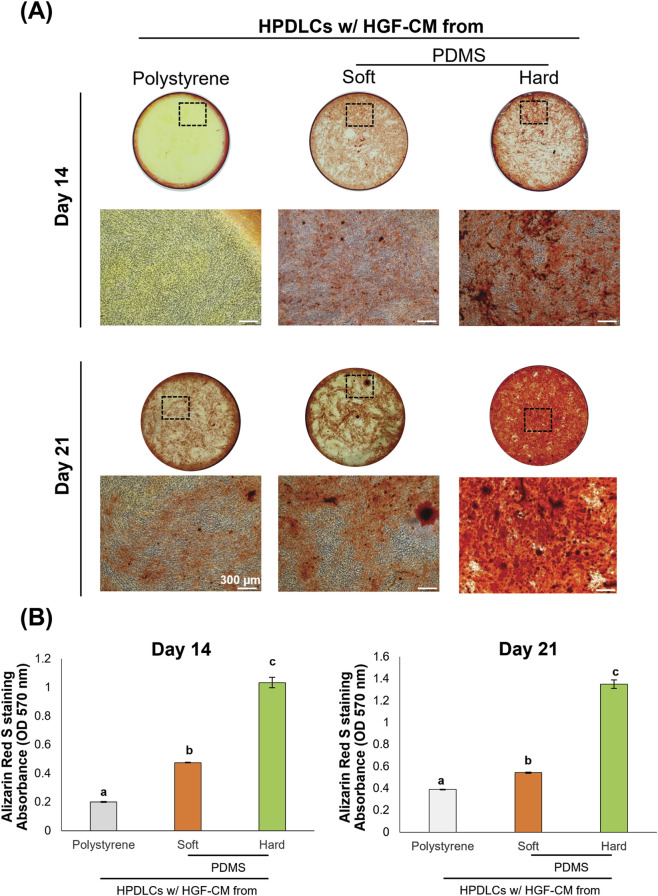
Effects of conditioned media of human gingival fibroblasts (HGF-CM) on substrate stiffness regulated mineralization of human periodontal ligament cells (HPDLCs). **(A)** Representing images of Alizarin Red S staining revealed mineralization (red stain) after osteogenic differentiation for 14 and 21 days. Dashed boxes in the macroscopic views indicate the specific regions corresponding to the high-magnification images. **(B)** The quantification of Alizarin Red S extracted from the stained cultures, measured by absorbance at 570 nm. Data were statistically analyzed by one-way ANOVA followed by Tukey’s multiple comparison tests (n = 3: P < 0.05). Data are presented as the mean ± standard deviation (SD), with different letters indicating statistically significant differences between multiple groups. The scale bar presents 300 μm. PDMS; polydimethylsiloxane.

Gene expression analysis revealed that HGF-CM from the hard substrate group significantly enhanced osteogenic marker expression compared with HGF-CM from other substrate groups. At day 14, both *ALP* and *OPN* were significantly upregulated in the HGF-CM from hard PDMS. By day 21, osteogenic markers including *ALP*, *RUNX2*, *COL1A1*, *OCN*, and *OSX* were all significantly elevated under HGF-CM from hard substrate conditions (Figure 5A). In addition, immunofluorescence staining revealed stronger OSX expression in HPDLCs cultured with HGF-CM from the hard substrates compared with those from soft substrates (Figure 5B). Alizarin red S staining demonstrated more intense mineral deposition in the HGF-CM from hard substrate at both time points (Figure 6A). The quantitative data confirmed these findings, revealing a statistically significant increase in calcium mineralization in the hard-substrate HGF-CM group compared to both the soft-substrate and polystyrene groups (Figure 6B).

### 3.6 Inhibition of CXCR4 attenuated the stiffness-mediated osteogenic effects of HGF-CM on HPDLCs

**FIGURE 7:**
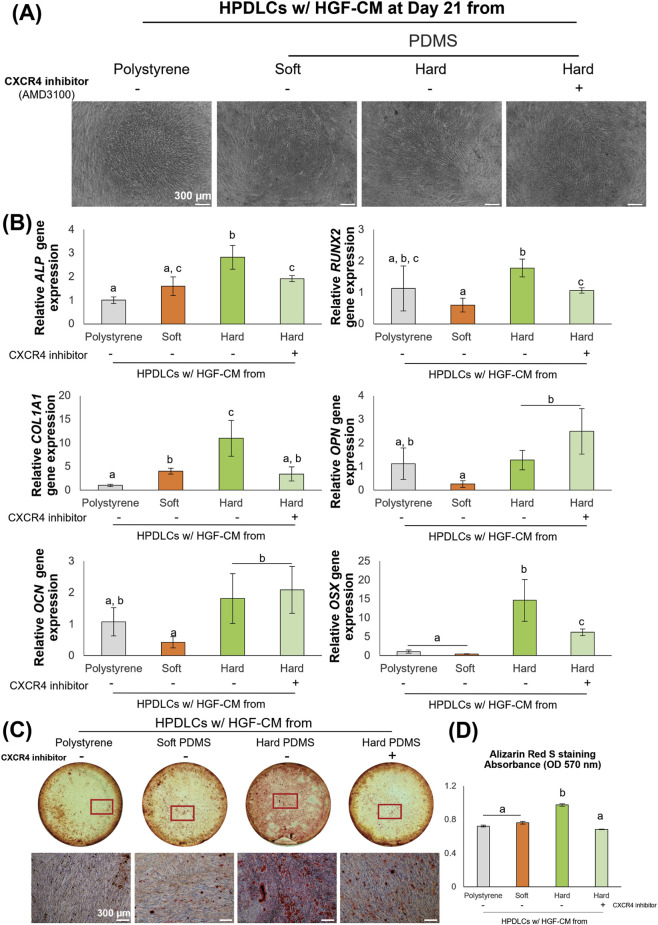
Inhibition of CXCR4 attenuated the stiffness-mediated osteogenic effects of HGF-CM on HPDLCs **(A)** Human periodontal ligament cells (HPDLCs) cultured with HGF-CM in osteogenic medium with CXCR4 treatment. **(B)** Representing images of Alizarin Red S staining revealed mineralization (red stain) after osteogenic differentiation for 21 days. Dashed boxes in the macroscopic views indicate the specific regions corresponding to the high-magnification images. **(C)** The quantification of Alizarin Red S extracted from the stained cultures, measured by absorbance at 570 nm. **(D)** The quantification of Alizarin Red S extracted from the stained cultures, measured by absorbance at 570 nm. Data were statistically analyzed by one-way ANOVA followed by Tukey’s multiple comparison tests (n = 3: P < 0.05). Data are presented as the mean ± standard deviation (SD), with different letters indicating statistically significant differences between multiple groups. The scale bar presents 300 μm. PDMS; polydimethylsiloxane.

To functionally confirm the mechanism related the CXCL12/CXCR4 signaling, HPDLCs were treated with the CXCR4 inhibitor, AMD3100, prior to co-culture with HGF-CM from different substrates. The cell attachment remained unaffected following inhibitor treatment and throughout the co-culture period (Figure 7A). CXCR4 inhibition significantly downregulated the osteogenic markers *ALP*, *RUNX2*, *COL1A1*, and *OSX* in HPDLCs cultured with HGF-CM from the hard substrates compared to those with untreated inhibitor. However, the expression levels of *OPN* and *OCN* were not significantly altered (Figure 7B). Furthermore, Alizarin Red S staining demonstrated that HPDLCs cultured in HGF-CM from hard substrates exhibited a decrease in calcium mineralization following CXCR4 inhibitor treatment (Figures 7C,D). These results indicate that HGF-mediated osteogenic differentiation of HPDLCs associated with CXCR4 receptor.

## 4 Discussion

The physiological relevance of substrate stiffness is a factor in determining fibroblast behavior within the periodontal microenvironment. In the present study, we utilized PDMS substrates with stiffness values of 4.4 kPa and 26.2 kPa to model the mechanical diversity observed across different gingival phenotypes. The stiff substrate was specifically selected to mimic the thick gingival phenotype, characterized by a robust, collagen-dense ECM, as the physiological stiffness of healthy gingival tissue is approximately 20 kPa (Šimoliūnas et al., 2021). In contrast, the 4.4 kPa substrate serves as a model for the thin gingival phenotype or soft gingival tissue (Tiskratok et al., 2023). These results suggested that ECM stiffness acts as a physical cue that modulates the paracrine behavior of fibroblasts.

Our findings demonstrated that HGFs exhibited a notable response to increased substrate stiffness, characterized by the upregulation of IL-4 and IL-10 (Figure 1A), cytokines associated with anti-inflammatory and tissue reparative responses (Nikovics et al., 2023). This observation aligned with previous reports demonstrating that mechanical cues can influence inflammatory signaling in fibroblasts and other stromal cell populations (Rojasawasthien et al., 2025; Chen et al., 2022). Importantly, these findings demonstrated that the relationship between the biomechanical characteristics of the extracellular matrix and cellular responses within an immunomodulatory environment. Furthermore, substrate stiffness also influences the inflammatory responses of HGFs under inflammatory conditions. Consistent with previous reports (Tiskratok et al., 2023), HGFs cultured on stiff substrates showed reduced *MMP9* expression and increased *TIMP1* expression following lipopolysaccharide (LPS) stimulation, indicating that substrate stiffness modulated matrix metalloproteinase regulation even during inflammation (Figure 2B). Our findings aligned with previous research in hepatic stellate cells, indicating that fibrotic rigidities lead to a decrease MMP9 expression and secretion, while simultaneously increasing the secretion of TIMP-1 (Lachowski et al., 2019).

**FIGURE 8:**
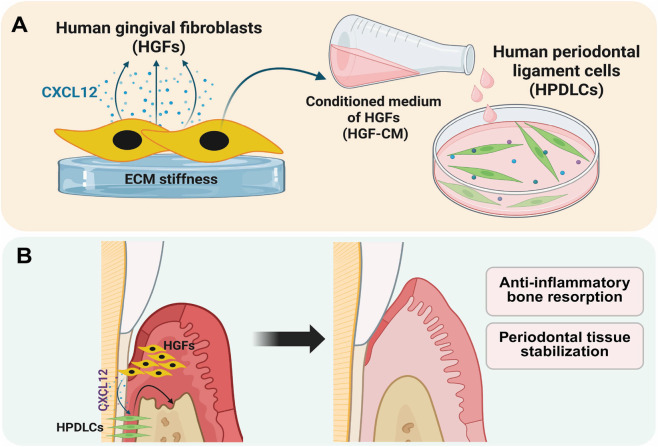
Schematic illustration shows the role of human gingival fibroblast–conditioned media (HGF-CM) in regulating osteogenic differentiation of human periodontal ligament cells (HPDLCs). **(A)** ECM stiffness stimulates CXCL12 chemokine expression in HGFs, which is associated with enhanced osteogenic responses in HPDLCs. **(B)** A potential clinical application of this mechanism is the utilization of HGF-derived factors to suppress inflammatory bone resorption and stabilize periodontal tissues. CXCL12: CXC motif chemokine 12.

Regarding the interplay between HGFs and HPDLCs in periodontitis progression, activated HGFs produce elevated levels of cytokines, chemokines, and matrix metalloproteinase, which influence inflammatory response and connective tissue breakdown (Wielento et al., 2023). Conversely, fibroblast-derived chemokines support tissue remodeling and osteogenic responses during tissue regeneration by recruiting and activating mesenchymal progenitor cells (Wang et al., 2024; Lin et al., 2017). In this study, we specifically focused our investigation on the chemokine CXCL12 because of its constitutively high expression in HGFs (Hosokawa et al., 2005; Morandini et al., 2013) and its well-established role in promoting the proliferation, angiogenesis, and osteogenic differentiation of HPDLCs (Zhang et al., 2017; Roato et al., 2025; Gao and Wu, 2024). Furthermore, our results demonstrated that HGFs cultured on stiff substrates exhibited increased CXCL12 expression and the corresponding conditioned media were associated with increased CXCR4 expression and osteogenic marker expression in HPDLCs (Figure 8). Importantly, targeted functional blocking with a CXCR4 inhibitor significantly reversed these osteogenic effects, providing direct evidence for the causal involvement of the CXCL12/CXCR4 signaling. These findings indicated that a stiffness-associated correlation between fibroblast paracrine output and HPDLC phenotype. Collectively, these findings support a model in which substrate stiffness alters the fibroblast paracrine environment, which in turn is associated with alteration in HPDLC behavior (Ramadan et al., 2020; Zhao et al., 2025).

HGF-CM from hard substrates was associated with an enhanced the osteogenic potential of HPDLCs, as indicated by the upregulation of osteogenic markers at both day 14 and 21. Previous studies have attributed similar effects to differential substrate stiffness regulating osteogenesis via ERK-mediated YAP nuclear translocation (Fu et al., 2025). In our observations, OPN and ALP were strongly expressed early, while sustained elevation of OSX and OCN at day 21 suggested long-term commitment to the osteoblastic lineage (Figures 5A,B). These transcriptional changes were further supported by Alizarin red S staining, where the HPDLCs treated with HGF-CM from hard substrates exhibited the most intense mineral deposition, indicating functional maturation into matrix-secreting osteoblasts. This observation emphasized the influence of the overall HGF-derived secretome on mineralization.

In this study, CXCL12, a chemokine typically associated with stem cell recruitment and osteogenesis (Verheijen et al., 2020; Gilbert et al., 2019), showed significantly higher expression in HGFs cultured on hard substrates compared to those on soft substrates (Figures 1C,D). This indicated that substrate stiffness influenced alteration of the HGF secretome. The regulation of CXCL12 by substrate stiffness was associated with p38 MAPK activity, as pharmacological inhibition of p38 MAPK with SB203580 reduced both CXCL12 gene expression and protein secretion in HGFs on stiff substrates (Figures 3A,B). These findings were consistent with previous studies demonstrating that p38 MAPK signaling were involved in stiffness-induced gene regulation in fibroblasts and stem cells (Allen et al., 2012; Selig et al., 2020). However, p38 MAPK activity are related to CXCL12 expression in HGFs, ERK inhibition had no significant effect on CXCL12 secretions in the stiffness-responsive behavior of these fibroblasts.

Furthermore, conditioned media derived from HGFs on hard substrates were associated with the upregulation of CXCR4, which is the primary receptor for CXCL12 in HPDLCs (Bernhagen et al., 2007). Previous studies have suggested that CXCL12/CXCR4 signaling is involved in progenitor cell activity (Shahnazari et al., 2013; Rodríguez-Carballo et al., 2016), with mechanical stimuli often regulating CXCL12 expression to support migratory responses, cell proliferation, and chemotaxis (Yuan et al., 2012) (Liang et al., 2021). In alignment with these studies, our functional blockade utilizing the CXCR4 inhibitor directly attenuated the stiffness-induced osteogenic differentiation of HPDLCs *in vitro*, confirming that CXCL12/CXCR4 signaling actively mediates this paracrine interaction (Figures 7B–D). Furthermore, inhibition of CXCR4 has been reported to attenuate osteogenic differentiation in stem cell precursors (He et al., 2019; Qin et al., 2021). Therefore, CXCL12/CXCR4-associated signaling might be a representative example of the paracrine alteration.

Despite these findings, this study had the limitations in fully characterizing the relationship between ECM stiffness and the HGF paracrine landscape. Although our investigation specifically targeted the chemokine CXCL12, HGFs possess a highly complex secretome and release a diverse array of paracrine factors that influence neighboring cells (Williams et al., 2021; Smedas et al., 2025). Focusing on a single chemokine provides an incomplete representation of the mechanosensitive microenvironment. Therefore, future studies utilizing high-throughput omics profiling of the conditioned medium are necessary to fully map this broader secretome. Moreover, periodontal tissue consists of heterogeneous cell populations, including other resident immune and stem cells; for instance, substrate stiffness has been shown to modulate macrophage inflammatory responses (Meli et al., 2020), suggesting that the response of resident immune cells to stiffness warrants future research. Additionally, while the p38 MAPK activity and CXCL12/CXCR4 findings were associated with the stiffness-responsive behavior of HGFs and HPDLCs, definitive signaling pathway was not directly tested via blockade experiments or inhibitor treatment. Therefore, these pathways should be viewed as representative examples of the broader paracrine alteration, rather than a definitive signaling. Furthermore, to fully substantiate how mechanotransduction regulates periodontal homeostasis, biochemical and functional analyses in three-dimensional or animal models, where stiffness can be modulated independently of inflammation, are required. Nevertheless, this study proposed the relationship between ECM stiffness and the HGF paracrine landscape, a phenomenon that might be involved in the high susceptibility for periodontal tissue homeostasis. These results support the clinical significance of the gingival phenotype and offer a basis for further exploration in anti-inflammatory response and periodontal tissue stabilization (Figure 8B).

## 5 Conclusion

Substrate stiffness modulates the paracrine behavior of human gingival fibroblasts, with CXCL12 serving as one representative example of a stiffness-responsive factor. Stiffness-associated changes in the gingival fibroblast secretome were associated with altered osteogenic and inflammatory responses in human periodontal ligament cells. These findings highlight the influence of the physical microenvironment on fibroblast–periodontal ligament cell interactions and provide a basis for future mechanobiology-focused studies on periodontal tissue stabilization.

## Data Availability

The datasets presented in this study can be found in online repositories. The names of the repository/repositories and accession number(s) can be found in the article/Supplementary Material.
