# Supplementary material for: Substrate stiffness modulates human gingival fibroblast paracrine signaling to promote osteogenic differentiation of human periodontal ligament cells
Source: Front Bioeng Biotechnol. 2026 Apr 15;14:1753774. doi: 10.3389/fbioe.2026.1753774 (PMC13126313; doi:10.3389/fbioe.2026.1753774)
Supplement: Supplementary file 1 [file Supplementaryfile1.docx]

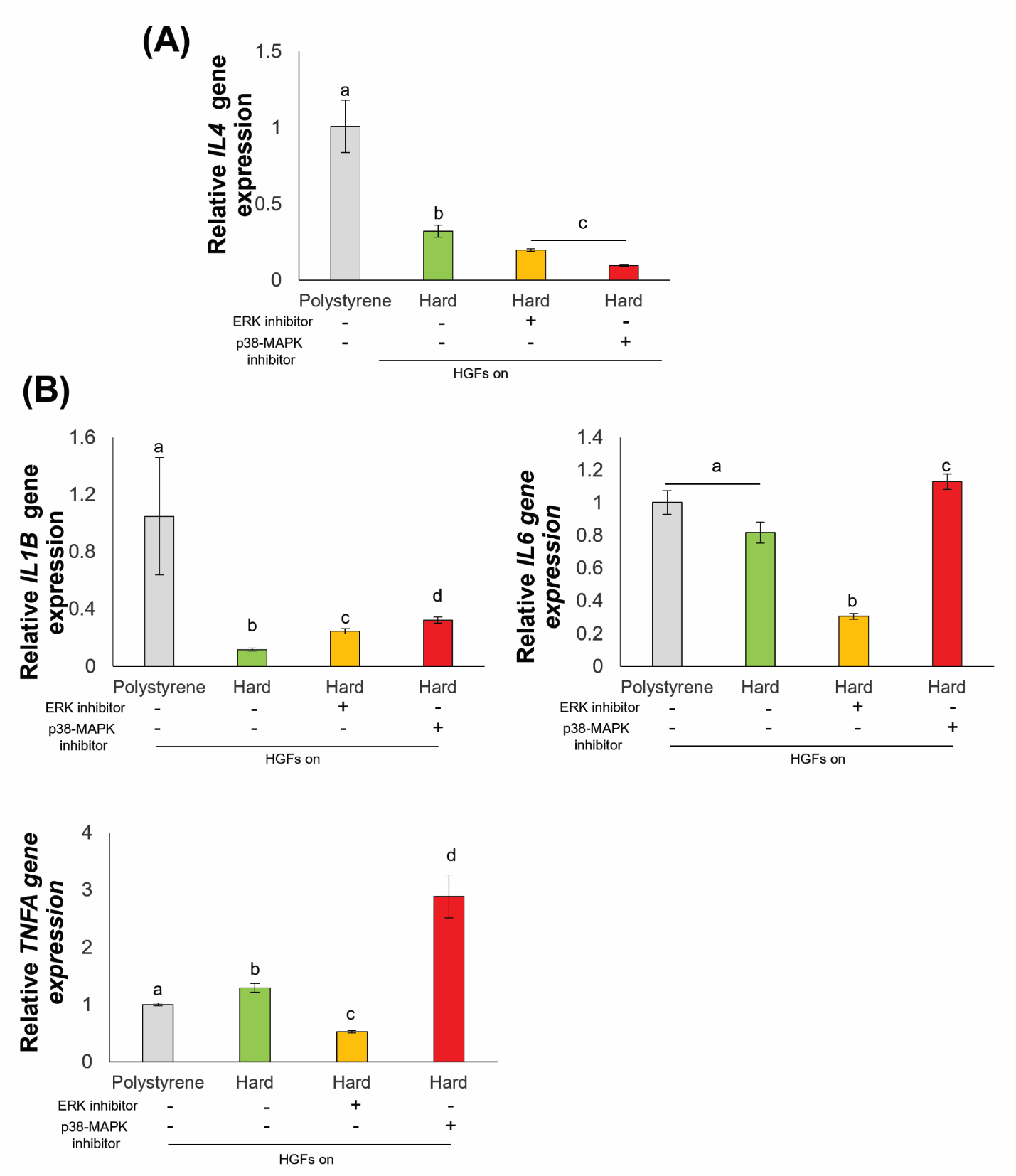


**Supplementary Figure 1**. Mitogen-activated protein kinase (MAPK) pathway regulated substrate stiffness-induced pro- and anti-inflammatory expression in human gingival fibroblasts (HGFs). Real-time RT-PCR was performed to detect gene expression levels of **(A)** *IL4*, **(B)** *IL1B*, *IL6*, and *TNFA*. The expression of GAPDH was used as an internal control. Data were statistically analyzed by one-way ANOVA followed by Tukey’s multiple comparison tests (n = 3: P < 0.05).
